# TELERA—Asynchronous TELEmedicine for Patients With Rheumatoid Arthritis: Study Protocol for a Prospective, Multi-Center, Randomized Controlled Trial

**DOI:** 10.3389/fmed.2021.791715

**Published:** 2021-12-13

**Authors:** Johanna Mucke, Johannes Knitza, Felix Muehlensiepen, Manuel Grahammer, Ramona Stenzel, David Simon, Arnd Kleyer, Gerhard Krönke, Charlotte Sharp, Gerlinde Bendzuck, Marianne Korinth, Corinna Elling-Audersch, Nicolas Vuillerme, Georg Schett, Ann-Christin Pecher, Martin Krusche

**Affiliations:** ^1^Policlinic and Hiller Research Unit for Rheumatology, Medical Faculty and University Hospital Düsseldorf, Heinrich Heine University Düsseldorf, Düsseldorf, Germany; ^2^Department of Internal Medicine Rheumatology and Immunology, Friedrich-Alexander- University Erlangen-Nürnberg (FAU), Universitätsklinikum Erlangen, Erlangen, Germany; ^3^Deutsches Zentrum fuer Immuntherapie (DZI), FAU Erlangen-Nuremberg and Universitätsklinikum, Erlangen, Germany; ^4^Université Grenoble Alpes, AGEIS, Grenoble, France; ^5^Center for Health Services Research, Brandenburg Medical School Theodor Fontane, Ruedersdorf, Germany; ^6^Faculty of Health Sciences Brandenburg, Brandenburg Medical School Theodor Fontane, Neuruppin, Germany; ^7^Abaton GmbH, Berlin, Germany; ^8^Centre for Epidemiology Versus Arthritis, Division of Musculoskeletal and Dermatological Sciences, School of Biological Sciences, The University of Manchester, Manchester, United Kingdom; ^9^Deutsche Rheuma-Liga Bundesverband e.V., Bonn, Germany; ^10^LabCom Telecom4Health, Orange Labs and Univ. Grenoble Alpes, CNRS, Inria, Grenoble INP-UGA, Grenoble, France; ^11^Institut Universitaire de France, Paris, France; ^12^Centre for Interdisciplinary Clinical Immunology, Rheumatology and Autoinflammatory Diseases, University Hospital Tuebingen, Tübingen, Germany; ^13^Division of Rheumatology and Inflammatory Rheumatic Diseases, University Hospital Hamburg Eppendorf, Hamburg, Germany

**Keywords:** telemedicine, mHealth, remote care, rheumatoid arthritis, ePRO, digital health applications, mobile medical apps

## Abstract

Innovative strategies are needed to adequately assess and monitor disease activity of patients with rheumatoid arthritis (RA) in times of scarce appointments. The aim of the TELERA study is to evaluate the feasibility and performance of asynchronous telemedicine visits based on patient-generated data and patient's drug history. RA patients use a medical app, ABATON, that captures the results of a self-performed quick CRP-test, joint-count, and electronic patient-reported outcomes in between visits. This is a prospective, multi-center, randomized controlled trial performed in four German university centers. The estimated sample size is 120 patients. The main outcome is the agreement of rheumatologists' treatment decisions based on asynchronous telemedicine patient-generated data with traditional in-person rheumatology clinic-based decisions and with patient suggestions. The TELERA trial will provide evidence regarding the implementation of remote care in rheumatology.

**Clinical Trial Registration:** This clinical trial was registered at German Registry for Clinical Trials (DRKS). http://www.drks.de/DRKS00016350, identifier: DRKS00024928.

## Introduction

The treat-to-target concept has been established as a treatment principle for rheumatoid arthritis (RA) (1). The aim of this strategy is to define a treatment target at therapy initiation and to closely monitor treatment response in order to identify insufficient treatment success and modify the therapeutic strategy as needed. This approach represents a challenge for rheumatologists as frequent, one to three-monthly assessments are recommended in patients with active disease and resources are limited (1–3). In reality, therapies are not assessed frequently enough and, therefore, are all too often not adjusted to the current state of the disease (4). At least two important reasons for the currently poor disease management are (A) the limited access to rheumatology specialists and (B) an increasing follow-up appointment deficit for already diagnosed patients. This situation is likely to worsen as the current shortage of rheumatologists in Germany and other nations will probably increase even further in the future (5, 6).

Digitalization promises new ways to improve patient care and shape a more efficient and transparent health care environment (7, 8) and the large majority of German patients with rheumatic and musculoskeletal diseases (RMD) regularly uses a smartphone (9). Electronic patient reported outcomes (ePROs) and wearables facilitate continuous digital patient monitoring (“tight control”) (9, 10). By using telemedicine, patient care could be individually adapted according to disease activity and follow-up preferences (“virtual treat-to-target”) (11). By actively involving the patient in disease monitoring, self-efficacy and activation (“patient empowerment”) can be increased (12) and ultimately remission reached earlier (13). Furthermore, the use of telemedicine could enable the rheumatologist to work more flexibly and efficiently.

The aim of this study is to evaluate the feasibility and performance of asynchronous telemedicine visits based solely on patient-generated data in RA patients.

## Methods

### General Study Design

This is a prospective, multi-center, randomized controlled trial. This study has received ethical approval by the Ethical Committee of the University Clinic of Tuebingen (# 4442020). We aim to include 120 patients with RA and written informed consent, treated at the rheumatology outpatient units of the university hospitals Duesseldorf, Erlangen, Hamburg and Tuebingen (Germany).

**Figure 1:**
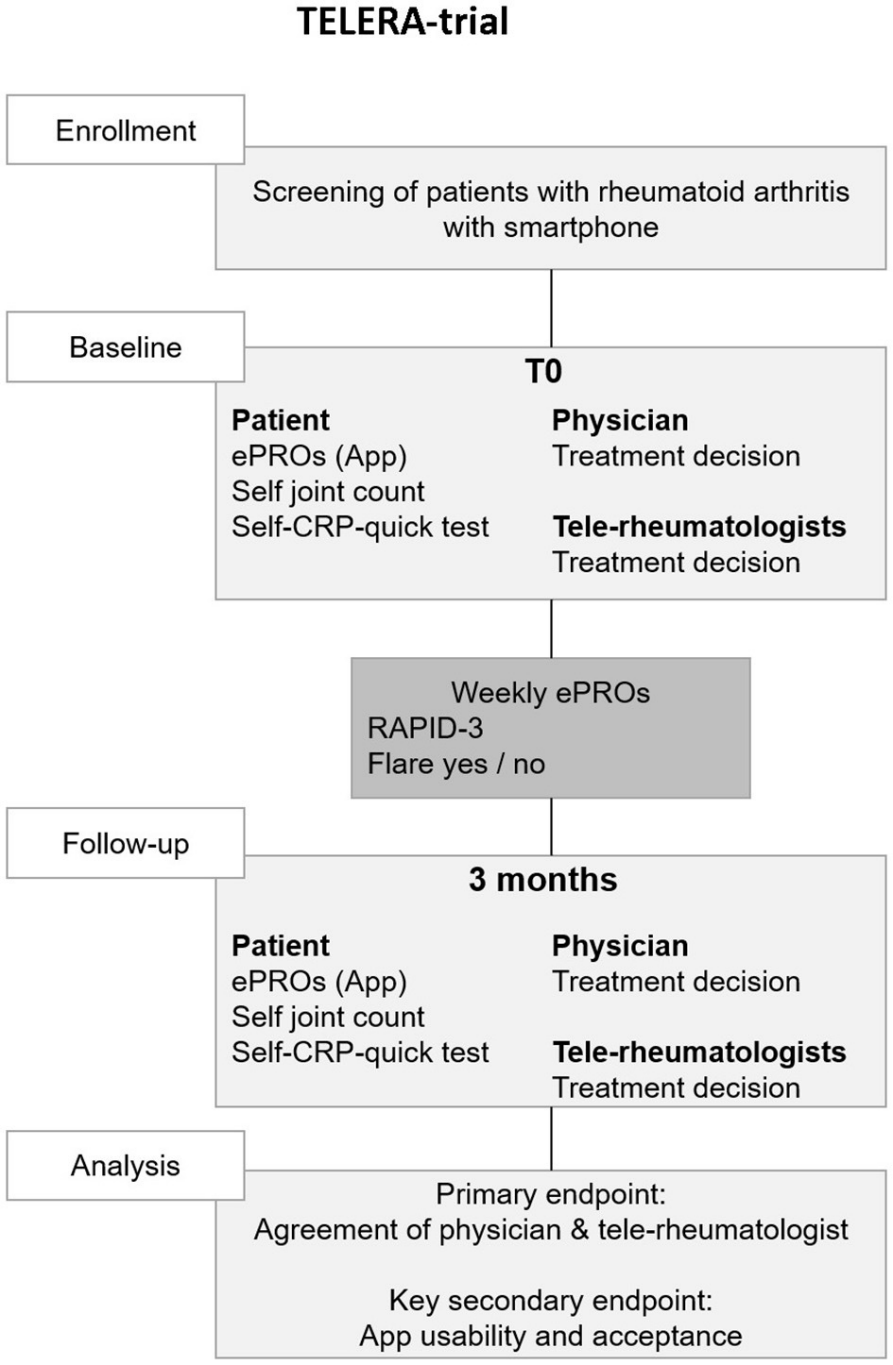
Trial flow chart. CRP, c-reactive protein; ePRO, electronic patient reported outcomes; RAPID-3, routine assessment of patient index data 3.

The overall study design is summarized in Figure 1. Prior to a regular face-to-face appointment (T0), patients with RA will be supported to carry out a structured self-examination of joints by a video and instructions from the study personnel and to perform a quick self C-reactive protein (CRP)-test. The patients will then enter the results in a study app. Furthermore, patients will be asked to answer ePROs on a weekly basis in between the regular next face-to-face appointment 3 months later (T1), when the self-examination will be repeated.

At each self-examination, the patient also enters his/her therapeutic suggestion: (A) escalation of therapy; (B) no therapeutic change needed; and (C) de-escalation of therapy. Based on patient history, the patient-generated data from T0, T1 and in-between visits, two independent randomized rheumatologists from other university centers will be asked to enter their therapeutic suggestion solely based on the digital information. The patient and tele-rheumatologists' suggestions are then compared to the gold standard which is the traditional therapeutic decision based on the face-to-face visit for T0 and T1.

After T1, patients complete an evaluation questionnaire and semi-structured telephone interviews will be conducted with 10 patients with RA and the four main study investigators to capture qualitative feedback concerning patient acceptance, app compliance, changes in self-management and self-efficacy.

### Patient Inclusion and Exclusion Criteria

Inclusion criteria are (1) the established diagnosis of RA according to the 2010 ACR/EULAR classification criteria; (2) > 18 years of age; (3) sufficient German language skills; (4) confidence in using a smartphone; and (5) written informed consent. Patients with newly diagnosed RA, unable to use a smartphone/tablet or to give written informed consent will be excluded from the trial. Eligible outpatient RA patients will be informed about the study by their local rheumatologist. If consent is given, they will be contacted by the respective trial physician (JK, JM, ACP, MK), invited to discuss the study, undergo screening for eligibility and sign the informed consent form.

### Study Visit

Prior to the routine clinical visits, patients are instructed to carry out a structured self-examination. This includes a self-assessed joint count, capillary-based CRP self-sampling, completion of ePROs and data entry into the study app. The duration of the study visit will be ~1 h.

### Joint Self-Examination

**Figure 2:**
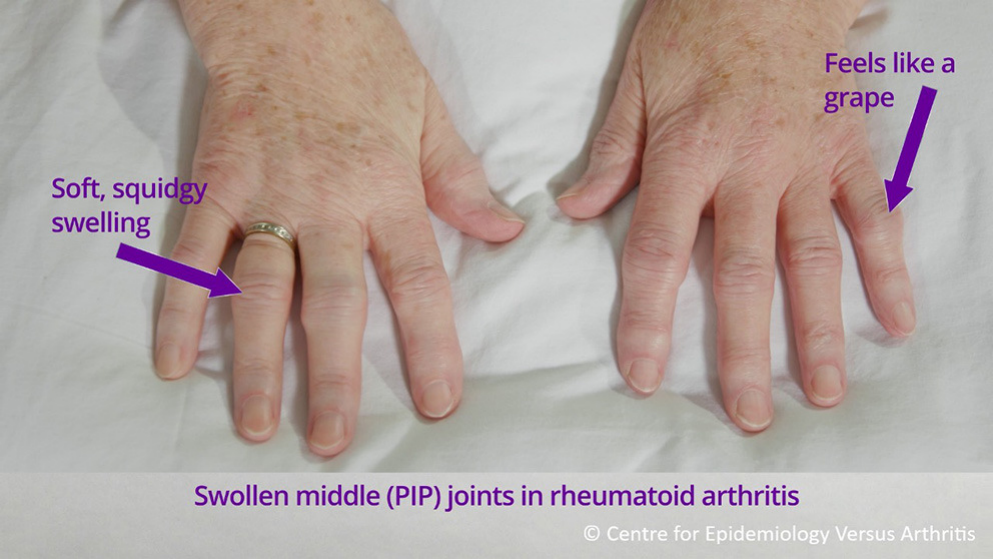
Instructions for self-examination of proximal interphalangeal joints (PIP), extracted from self-examination video.

The local study team teaches participants how to self-examine for tender and swollen joints using an instructional video (14). The video was co-developed with patients for the REMORA study (see Figure 2) (15). The video was translated to German by JK and dubbed using artificial intelligence by the commercial dubbing company Papercup^®^. The introduction provides an overview of how to correctly identify the joints included in the DAS28 score, including the differences between tender and swollen joints in rheumatoid arthritis vs. other conditions, and how to self-examine. In the video a patient is coached through self-examination by a nurse consultant, who then answers the patients' questions. The video is available open-access online in German (bit.ly/3rlYZTQ) and in English (bit.ly/3rlYZTQ), with optional subtitles. Any questions will be answered by the local study team, if needed.

### CRP Self-Sampling

**Figure 3:**
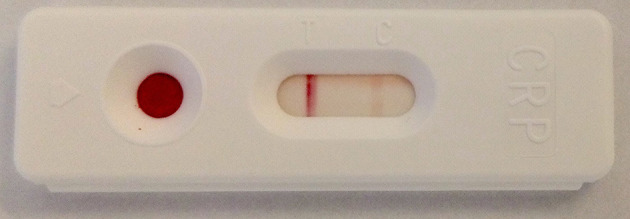
Example of a negative CRP test (T-line is more intense in color than C-line).

Patients receive written instructions on how to perform a semi-quantitative capillary-based CRP-test (CRP-CHECK-1,VEDALAB, Alencon, France). Any questions will be answered by the local study team, if needed, however the test should be carried out independently by patients and study personnel will only intervene to prevent harm to patients. The immunochromatographic rapid test allows semi-quantitative detection of CRP with three possible outcomes: (A) negative; <5 mg/L; (B) borderline; 5 to 10 mg/L or (C) positive; >10 mg/L. The test is currently only licensed for professional use, however we hypothesized that the test could also be carried out successfully by patients after appropriate education. Figure 3 displays an example of a negative CRP test. Supplementary Figure 1 shows the translated and adapted manual and Supplementary Figure 2 the interpretation guide.

### Study App and ePROs

**Figure 4:**
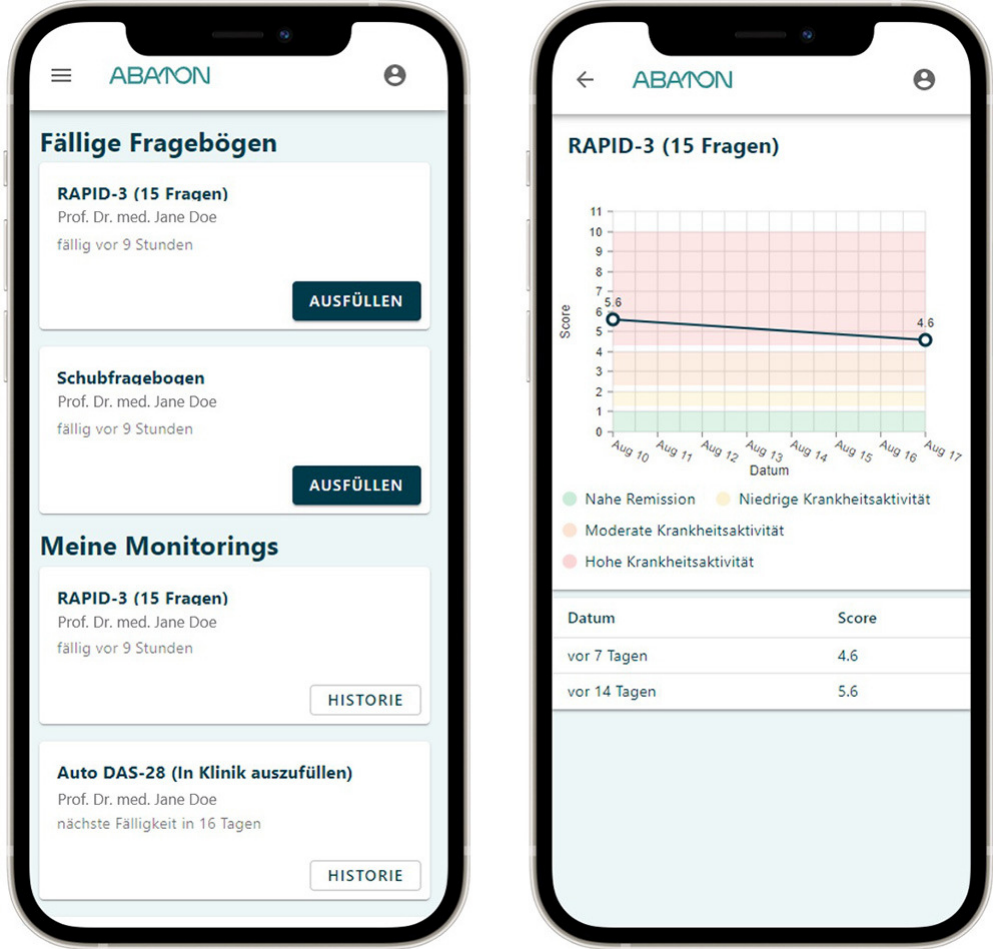
ABATON patient app screenshots, displaying questionnaire overview on the left and graphical display of RAPID-3 history on the right. RAPID 3 routine assessment of patient index data-3. Translations: Ausfüllen, Fill in; Datum, date; Fällige Fragebögen, due questionnaires; Fragen, questions; Historie, history; Hohe Krankheitsaktivität, high disease activity; In Klinik auszufüllen, to be answered in the outpatient clinic; Meine Monitorings, my monitorings; Moderate Krankheitsaktivität, moderate disease activity; Nahe Remission, close remission; Niedrige Krankheitsaktivität, low disease activity.

The ABATON web-app is a medical device developed and maintained by ABATON GmbH (Berlin, Germany). This app provides a study module which has been adopted to this trial. All digital administered questionnaires, forms and monitoring instruments were pre-configured. Each study center has access to the web-based study module via their own study account. Patients get invited by the respective clinical site study personnel via a short messaging system (SMS) invite which contains the personalized link for the patient. All questionnaires were administered on the patient's own phone (“bring your own device”). In addition to questionnaires completed by patients, all study relevant documentation (for example therapeutic suggestions of tele-rheumatologists) is performed digitally by the study personnel and patients. In addition to forms and questionnaires listed in Table 1, ABATON provides documentation for other parameters (Lab CRP, tender- and swollen joint count (TJC/SJC), medication information) and allows to add extra notes. In between visits, the patients are being asked to complete the RAPID-3 (routine assessment of patient index data 3) questionnaire and to state if they experienced a disease flare. If the flare question is answered with yes (“Did you experience a flare of your disease in the past 7 days?”), the patients are being asked to specify the duration (1–7 days) on a numeric rating scale with anchors (“On how many days did you experience the disease flare (in days)”). A reminder logic is implemented to remind the patients 3 consecutive days if they have not filled out the questionnaires and stop as soon as the due questionnaire is completed. Results are immediately available to the patient and the study site via the web-based dashboard. A graphical dashboard for the frequently entered questionnaires/scores is available to the patient all the time (see Figure 4). After having completed the T1 visit, patients will receive questionnaires to evaluate the app and the study experience via the study app after being manually triggered by the study personnel.

**Table 1:** Overview of questionnaires and data entered into ABATON software by patients, local rheumatologists and tele-rheumatologists.

**Name**	**Assessment**	**Filled by**	**Frequency**	**No. questions**
RAPID-3	Physical function, pain and global health	Patient	Weekly	15
Flare question	flares	Patient	Weekly	1–2 (two if patient experienced flare)
Auto-DAS-28 (CRP)	Disease activity	Patient	T0 and T1	5
PAM-13	Patient activation	Patient	T0 and T1	13
TELERA-set		Patient	T0 and T1	5
System-usability-scale	Usability and acceptance of ABATON app	Patient	T1	10
Net-Promoter-Score	Willingness of patients to recommend ABATON app	Patient	T1	1
Medical history	Disease duration, current and prior treatments, CRP-value.	Local rheumatologist	T0	3
Treatment question	Treatment decision (escalation, de-escalation or no change)	Local rheumatologist, Tele-rheumatologist 1, Tele-rheumatologist 2	T0 and T1	3

*CRP, c-reactive protein; PAM-13, patient activation measure 13; RAPID-3, 3 routine assessment of patient index data-3*.

### Routine Clinical Assessment

Local rheumatologists perform the routine patient visit including DAS-28 joint count, standard venous blood withdrawal for CRP analysis, recording of patient-perceived disease activity (VAS 0–10) and physician global assessment (PGA) (VAS 0–10).

### Data Collection and Web-Based Study Module

**Figure 5:**
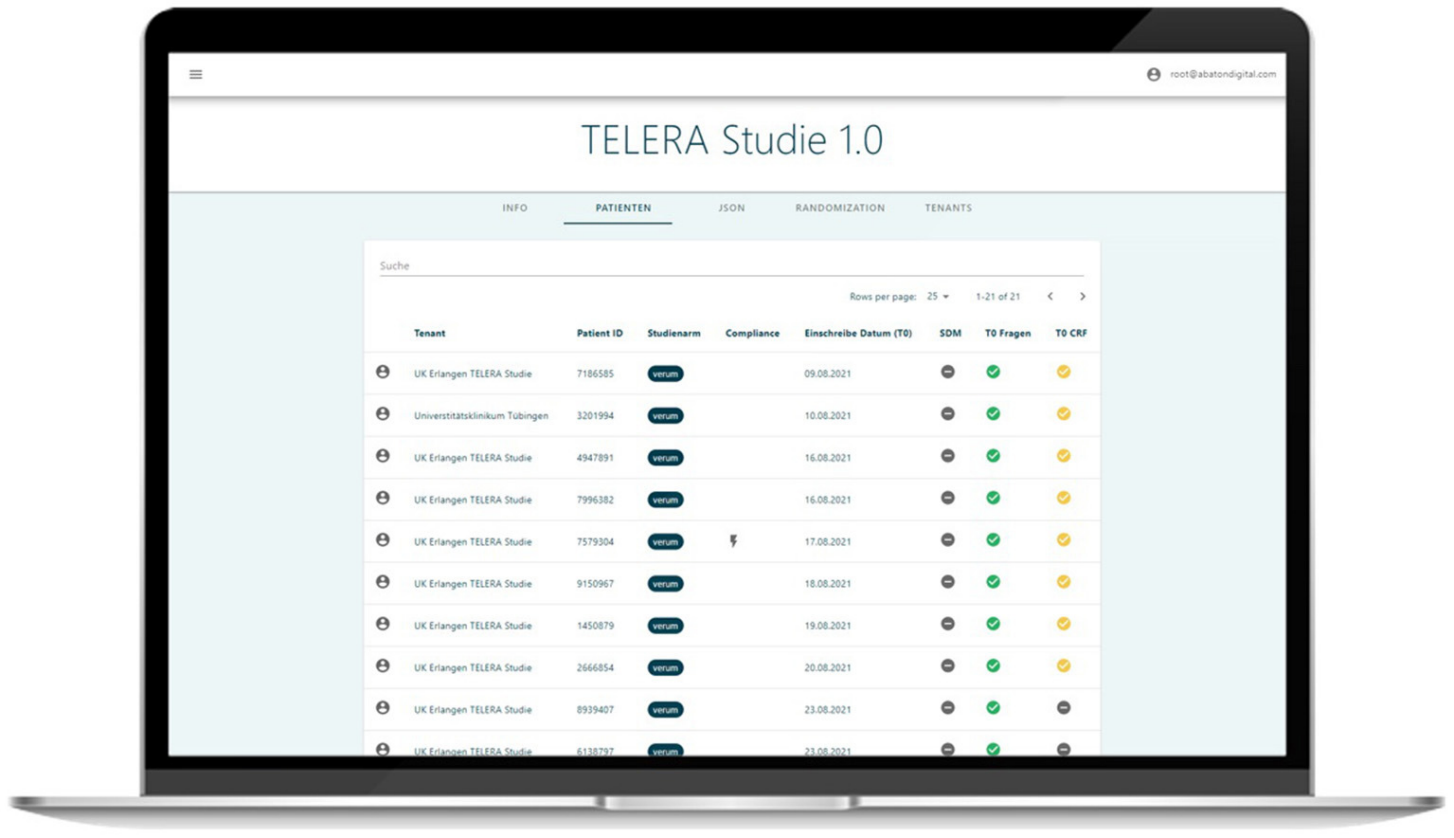
Screenshot of web-based study module displaying overview of the patients' statuses.

Data is collected using the ABATON platform with the study module (Figure 5) and the web-based patient app, which allow intuitive and rapid data entry. In addition to the patient-entered data via the application, general patient-related clinical data such as disease duration, previous and current medication is added manually to the ABATON platform by the local study personnel (Table 1). Furthermore, all rheumatologists (local rheumatologist and two tele-rheumatologists) enter their therapeutic decision for each patient and visit (T0 and T1) via the web-based application. ABATON allows a quick assessment of all study patients and the completeness status of related data points (monitoring, entries of health care professional, etc.).

### Randomization of Tele-Rheumatologists

The four participating rheumatologists (JM, JK, MK and ACP) are randomized to the respective cases T0 and T1 at the four centers, so that, for each patient visit (T0 and T1), therapeutic suggestions (escalate, no-change, deescalate) are available from two tele-rheumatologists by computer-generated block randomization with a block size of 2.

### Primary and Secondary Outcomes

Primary outcome will be the agreement of therapeutic suggestions of patients and tele-rheumatologists compared to the gold-standard, the therapeutic decision by the local rheumatologist.

Secondary endpoints will include: the overall usability and acceptance of the study app [measured by the System-Usability Scale by patients and physicians (SUS) and Net-Promoter Score (NPS)]; the agreement of patient- vs. physician-performed joint count; agreement of venous and capillary based CRP; willingness to perform CRP quick test among patients (NPS); travel time to rheumatologist (hours); agreement of tele-rheumatologist and local rheumatologist-based clinical disease activity index (CDAI); agreement of patient-based disease activity score (Auto-DAS28-CRP) and local rheumatologist-based DAS-28 CRP; change in patient's confidence for self-management (Patient Activation Measure, PAM) over time; willingness to substitute routine appointments for telemedicine appointments among patients (NPS); change of perceived assessment accuracy of rheumatologists and patients concerning patient disease activity since the last appointment (VAS 0-10).

### Sample Size

Due to the exploratory character of the trial, no formal sample size calculation was performed. A number of *n* = 120 patients was estimated to be sufficient to assess the primary outcome. With only two study visits 3 months apart, the estimated dropout will be low. However, even a moderate dropout rate of 20% would lead to a number of 96 patients to be analyzed.

### Measures and Questionnaires

#### Auto-DAS28-CRP

To assess disease activity, the CRP-based DAS-28 will used (16). The DAS28-CRP is a composite score comprising the acute phase parameters CRP, the patient reported disease activity in terms of the patient global assessment (PtGA) as well as the TJC and the swollen joint count SJC. The PtGA is assessed by a verbally administered numeric rating scale ranging from 0 to 100, asking for the patient's disease activity in the past 7 days. TJC and SJC comprise 28 predefined joints and are usually performed by a trained physician (16). In this study, an Auto-DAS28-CRP will be performed: The DAS-28 subdomains TJC and SJC will be assessed by the patient himself and then entered into the app, together with the PtGA at T0 and T1 and the result of the semi quantitative test (positive/borderline/negative). For calculation purposes we will use a pragmatic approach and enter CRP values of 0 μg/mL for negative results, 6 μg/mL for marginal values and 20 μg/mL for positive values. The values were chosen considering the manufacturers thresholds and a meaningful impact on the DAS28-CRP calculation.

#### Clinical Disease Activity Index (CDAI)

As an alternative RA specific disease activity score, the CDAI was chosen as a CRP-independent and purely clinical index. The CDAI includes the 28 joint count examination, the PGA and the PtGA and is used both in clinical practice and in clinical trials. It has been shown to be valid an sensitive for the assessment of RA activity and treatment response (17).

#### Routine Assessment of Patient Index Data (RAPID-3)

The routine assessment of patient index (RAPID-3) is a validated patient-reported outcome measure (PROM) for RA patients (18), evaluating the patient's physical function, pain, and global health. It's electronic version is validated (19).

#### Patient Activation Measure (PAM)

The PAM13 is a 13-item measure that assesses patient activation, including their knowledge, skills and confidence to manage their own health and well-being (20). The 13 items are answered on a Likert scale from 1 to 4 (strongly disagree to strongly agree). This questionnaire reflects the four stages of activation. Level 1: Patients believe that their role is important (item 1 and 2); level 2: patients have the confidence and knowledge to take an active part (items 3–8); level 3: patients take action and participate actively (items 9–11); level 4: patients have integrated their active role into their everyday life and hold on to it in stressful situations (items 12–13). Higher PAM scores indicate higher patient activation. The validated German version PAM13-D will be used (21).

#### System-Usability Scale (SUS)

Usability and acceptance of the ABATON app will be tested using the SUS (22). The SUS consists of a 10-item questionnaire answered on a scale from 1 to 5 (strongly disagree to strongly agree) (22). The scores for each question are added and multiplied by 2.5 to transform to a 0–100 scale. Scores above 68 are considered above average (22).

#### Net-Promoter-Score (NPS)

The NPS measures the willingness of the participants to recommend the ABATON app to another patient (23). Participants answer using a 11-point numeric rating scale (0 not at all likely, 10 extremely likely). Answers between 0 and 6 are summarized as detractors, 7–8 as passives and 9–10 as promoters. The NPS is calculated by subtracting the percentage of detractors from the percentage of promoters (23).

### Semi-structured Telephone Interviews

To explore patient acceptance, app compliance, change in patient's confidence for self-management and change in patient's perceived self-efficacy, complementary qualitative interviews are conducted with patients (*n* = 10) and providers (*n* = 4). The interviews are held via telephone, using a semi-open interview guide. The interviews are audio-recorded, transcribed verbatim and analyzed using qualitative content analysis (24). To achieve intersubjectivity and consistency, the analytical work is carried out within the group for qualitative research of the Centre for Health Services Research Brandenburg using the analysis software MAXQDA.

### Statistical Analysis

Interrater reliability will be calculated by Cohen's kappa. For this exploratory study, 120 patients with RA will be recruited. Correlation analyses will be used to assess the agreement of patient- vs. physician-based DAS-28 and the clinical decisions of patient vs. tele-rheumatologists and face-to-face rheumatologist. Multivariable regression analyses will be performed to assess changes in self-management and self-efficacy and their influential factors over time. Analyses will be adjusted for potential confounders such as age, disease duration, disease activity and the number and type (conventional disease modifying antirheumatic drugs (DMARD), biological DMARD or targeted-synthetic DMARD) of previous treatments.

## Results

Enrolment of participants started in August 2021. Recruitment of 120 patients and follow-up assessments are expected to be completed by June 2022. Thus far, patient feedback has been positive. Patients seem to appreciate the ability to receive empowering in-depth information about disease evaluation and to independently assess their disease activity.

## Discussion

TELERA is an exploratory study to assess the feasibility and performance of asynchronous telemedical assessments of patients with RA. The overall aim is to move current rheumatology care toward a needs-based approach with the help of remote care. Currently, the number of new rheumatologists is declining (3) and it still takes multiple months for patients to see a rheumatologist (25). However, we could demonstrate that the majority of patients and rheumatologists agreed that patients in remission do not need to be seen in person (26). The recently published EULAR guidelines highlight the importance of self-management skills and potential of mobile apps to improve the clinical care of people with RA (27). Importantly, Thurah et al. could previously demonstrate in a RCT that a remote care approach was safe and not inferior to conventional management in RA (28). In this RCT, 294 patients with RA were requested to complete ePROs and a CRP measurement each 3–4 months. The CRP measurement was however not performed by patients themselves, no joint-count was carried out and ePRO questionnaires were completed before tele-health follow-ups, which were synchronous. Based on our previous work (9), we chose a 1 week ePRO frequency, providing rheumatologists and patients with continuous disease activity data and including self-sampling (26, 29). Furthermore, we wanted to see if our study confirms previous results showing a significant increase in patient activation (PAM) after using an RA monitoring app (12). The benefits of an intensive RA telemonitoring strategy have also been demonstrated by Salaffi et al. (13), who could show that patients with early arthritis reach the state of remission more often and faster when receiving frequent telemonitoring and treatment adjustments according to a standardized treatment protocol rather than standard of care. In our opinion, the simple shift from physical in-person visits to virtual visits will not result in a significant liberation of resources and in particular time of rheumatologists. Nevertheless, asynchronous visits give patients and rheumatologists the freedom of time and place. By using a combination of objective and subjective markers for disease-activity self-assessment, patients can inform their rheumatologists in detail about their health status. Based on this remote patient-generated information, rheumatologists should continuously adapt the follow-up strategy incorporating remote care strategies (30): in case a patient achieves remission after therapy induction, the subsequent ePRO frequency could be extended and a remote follow-up (i.e., telephone call) after 3 months could be scheduled instead of a face-to-face meeting. Web based asynchronous home telemonitoring is already an integral part in the care of other chronic diseases such as diabetes and heart disease (31), as well as inflammatory bowel disease (32) and has great potential in rheumatology (33). To the best of our knowledge, this is the first trial evaluating the potential of asynchronous telemedicine visits based on patient-based joint counts, self-performed CRP and ePROS in RA patients. Furthermore, it is the first study comparing treatment decisions of patients, rheumatologists and tele-rheumatologists. A major strength of this study is the early patient involvement. The study design and protocol was designed in close cooperation with three official patient research partners of the German League against Rheumatism (Deutsche Rheuma-Liga Bundesverband e.V.) who commented on and edited the draft version and approved the final study protocol. We deliberately chose a risk-adverse study setting, where participation has only a minimal impact on clinical care (rheumatologists have access to ePROS in-between visits). This study design has some limitations. Patients eligible for the study need to be in possession of a smartphone and need to have a sufficient level of understanding and dexterity to use the app, resulting in exclusion of some elderly or cognitively impaired, as well as underprivileged patients without smartphone or network access. This is a general limitation in app-based remote health care monitoring and will most probably change within the next decades. Similary, the chose time-frame limits the analysis to two appointments per patient.

Due to the exploratory study character, patients will not be stratified by disease duration, activity or previous treatments. Analyses will however be adjusted for these possible confounders. Furthermore subgroup analyses will be performed to assess potential differences between patients in remission or with active disease. Also, remote evaluation can be difficult in patients with recent treatment changes who have experienced some improvement but still have active disease. For these cases, remote rheumatologists receive information on previous therapies including recent changes.

The usage of semi-quantitative CRP tests provides only a certain degree of accuracy. However, this is a simple testing system that could be performed at home without the need of extensive equipment. Further, after discussing the options with patients, it seemed more important to receive approximate results in a short period of time than exact results after multiple days.

## Conclusion

Anticipating an aggravation of the current shortage of rheumatologists, we are convinced that it offers even greater value to empower patients to assess their disease activity more professionally and independently use quick CRP tests, ePROs and a self-performed joint count. Ultimately, this patient empowerment in combination with technological innovation could result in more need-adapted visits and increase of remote care.

## Ethics Statement

The studies involving human participants were reviewed and approved by Ethical Committee of the University Clinic of Tuebingen (# 4442020). The patients/participants will be required to provide written informed consent to participate in this study.

## Author Contributions

MK and JK developed the concept of the trial. JM ran the statistical analyses. MG developed, maintained, and adapted the ABATON study module and web-based application. CS developed the self-examination video, adapted for use in German by JK and A-CP. JM, JK, and A-CP drafted the manuscript which was revised by MK and JK. All authors contributed to the design of the trial. All authors contributed to the article and approved the submitted version.

## Funding

This project was supported by Sanofi-Aventis as the winner of the Sanofi GeneRAtion program. Sanofi had no role in the design of this study and will not have any role during its execution, analyses, interpretation of the data, or decision to submit results. This work was supported by the Deutsche Forschungsgemeinschaft (DFG—FOR 2886 PANDORA—B01/A03/Z01 to GK, GS, and AK).

## Conflict of Interest

JK, JM, A-CP, and MK have received research support from Sanofi. JK reports personal fees from ABATON GmbH. MG is founder and shareholder of ABATON GmbH. The remaining authors declare that the research was conducted in the absence of any commercial or financial relationships that could be construed as a potential conflict of interest.

## Publisher's Note

All claims expressed in this article are solely those of the authors and do not necessarily represent those of their affiliated organizations, or those of the publisher, the editors and the reviewers. Any product that may be evaluated in this article, or claim that may be made by its manufacturer, is not guaranteed or endorsed by the publisher.
