# Supplementary material for: TELERA—Asynchronous TELEmedicine for Patients With Rheumatoid Arthritis: Study Protocol for a Prospective, Multi-Center, Randomized Controlled Trial
Source: Front Med (Lausanne). 2021 Dec 13;8:791715. doi: 10.3389/fmed.2021.791715 (PMC8710736; doi:10.3389/fmed.2021.791715)
Supplement: Supplementary file 1 [file Data_Sheet_1.docx]

Supplementary Material

## Supplementary Figures

##
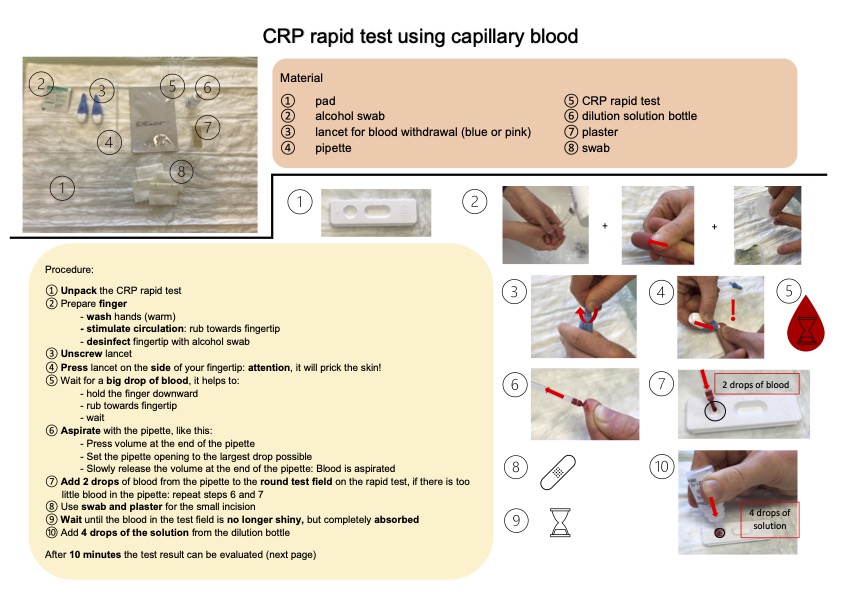


**Supplementary Figure 1.** Translated CRP patient self-sampling instructions. CRP c-reactive protein


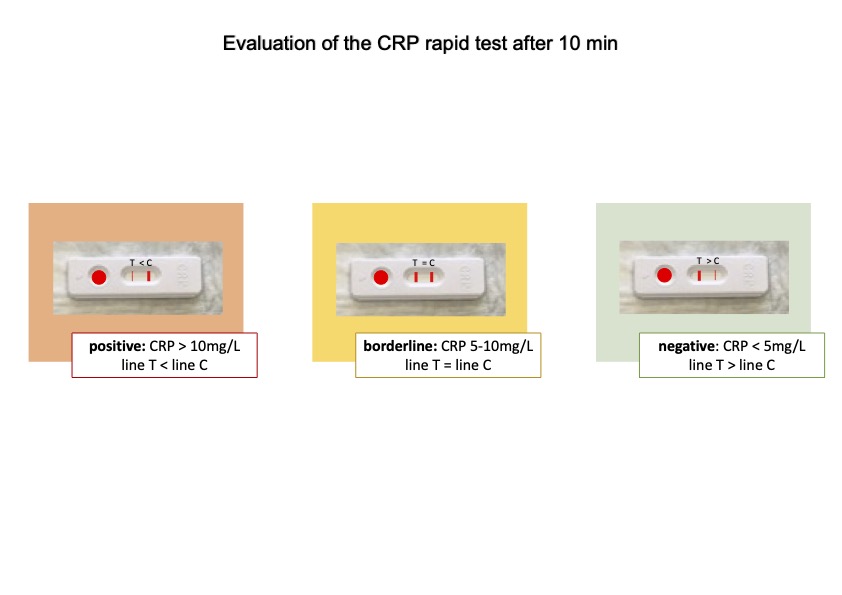


**Supplementary Figure 2.** Translated interpretation guide for CRP self-sampling test.
